# EAST (Epilepsy, Ataxia, Sensorineural Hearing Loss, and Renal Tubulopathy) Syndrome: A Rare Association Between Brain, Ear, and Kidney

**DOI:** 10.7759/cureus.68909

**Published:** 2024-09-07

**Authors:** Aditi Vats, Amit Satpathy, Biswajit Sahoo, Abhilash Sahoo, Manoj Kumar Nayak

**Affiliations:** 1 Radiodiagnosis, All India Institute of Medical Sciences, Bhubaneswar, Bhubaneswar, IND; 2 Pediatrics, All India Institute of Medical Sciences, Bhubaneswar, Bhubaneswar, IND

**Keywords:** ataxia, east syndrome, epilepsy, kcnj10, kir4.1, renal tubulopathy, sensorineural hearing loss

## Abstract

EAST syndrome - Epilepsy, Ataxia, Sensorineural hearing loss, and renal Tubulopathy - is an autosomal recessive disorder affecting the potassium channel in the brain, inner ear, and basolateral membrane of the distal nephron of the kidney. The mutation in the *KCNJ10 *gene is responsible for defective potassium transport in those locations, resulting in seizures, hearing loss, and hypokalemia. Imaging findings of this disease are typical, such as cerebellar hypoplasia and signal changes in bilateral dentate nuclei, midbrain, pons, and medulla, with variable restricted diffusion due to intramyelinic edema. Variable degrees of atrophy can be seen in the brainstem, spinal cord, corpus callosum, and cortex. No definitive treatment has been described yet in literature, and management is focussed mainly on symptomatic treatment like antiepileptics for seizures and potassium supplementations for hypokalemia. Although limited case reports are described in the literature, most reports described this as a non-progressive disorder. Herein, we describe a case of EAST syndrome in a three-year-old male child with a history of seizures, global developmental delay, bilateral sensorineural hearing loss, salt-wasting renal tubulopathy, and imaging of the brain showed diffuse cerebral atrophy with signal changes in the brainstem and bilateral dentate nuclei, showed clinical improvement on symptomatic management.

## Introduction

EAST syndrome (Epilepsy, Ataxia, Sensorineural hearing loss, and renal Tubulopathy) is an autosomal recessive disorder associated with potassium channelopathy with a prevalence of 1:1000,000 [1]. The underlying mutation is the *KCNJ10 *gene in chromosome 1, which encodes for Kir4.1 (an inwardly rectifying potassium channel), present variably in the inner ear, central nervous system, and basolateral membrane of distal nephrons of kidneys, ultimately leading phenotypic variations in symptoms and their severity [2]. Other salt-wasting renal tubulopathies involving distal nephrons, including Gitelman and Bartter syndromes, also have similar features of normotensive hypokalemic alkalosis. However, EAST syndrome has other extra-renal manifestations besides renal findings [3]. No definitive treatment has been described yet in the literature, and management is focused mainly on symptomatic treatment. Herein, we describe a case of EAST syndrome with typical clinical and imaging features with symptomatic management in a 3-year-old male child.

## Case presentation

A 3-year-old boy had a known case of seizure disorder diagnosed at the age of 3 months, which was a generalised onset tonic-clonic type. The seizure was partially controlled with intermittent episodes with two antiepileptics (Levetiracetam and valproate). On subsequent visits, he was diagnosed with global developmental delay, microcephaly, salt-wasting renal tubulopathy, and bilateral moderate sensorineural hearing loss. He was diagnosed with normotensive hypokalemic alkalosis at the age of 6 months and was started on medications for the same. His antenatal and perinatal history was unremarkable, except for low birth weight. General examination showed a global developmental delay of all the milestones. Central nervous system examination revealed reduced tone in all four limbs and power of 3/5 in bilateral upper and lower limbs with normal bulk. Cerebellar examination showed no nystagmus, titubation, and other cerebellar signs. His blood parameters were within normal limits except for persistent hypokalemia with metabolic alkalosis (Table 1).

**Table 1:** Different blood parameters of the patient with EAST syndrome EAST: Epilepsy, Ataxia, Sensorineural Hearing Loss, and Renal Tubulopathy Syndrome

Investigation	Value	Reference range
Haemoglobin (g/dL)	9.3	11.5-15.5
Total Leucocyte Count (/mL)	10,380	5500-15500
Neutrophils (%)	40	23-45
Lymphocytes (%)	54	35-65
Eosinophils (%)	00	0-3
Platelet count (/mL)	8,80,000	1,50,000-4,50,000
Peripheral blood smear	Microcytic and hypochromic red cells with few polychromatophils	
Mean Corpuscular Volume (fL)	50.6	75-87
Mean Cell Hemoglobin (pg/cell)	14	24-30
Haematocrit (%)	31.8	34-40
C-Reactive Protein (mg/L)	2.9	0-3
Blood urea (mg/dL)	16	9-23
Creatinine (mg/dL)	0.78	0.70-1.30
Sodium (mmol/L)	128	132-146
Potassium (mmol/L)	2.42	3.5-5.5
Chloride (mmol/L)	84	99-109
Uric acid (mg/dL)	0.5	3.5-7.2
Serum Total Bilirubin (mg/dL)	0.4	0.2-1.1
Serum Direct Bilirubin (mg/dL)	0.1	0-0.3
Aspartate Aminotransferase (U/L)	29	<34
Alanine Aminotransferase (U/L)	35	10-49
Serum Alkaline Phosphatase (U/L)	121	46-116
Serum Protein (g/dL)	7.7	5.7-8.2

**Figure 1:**
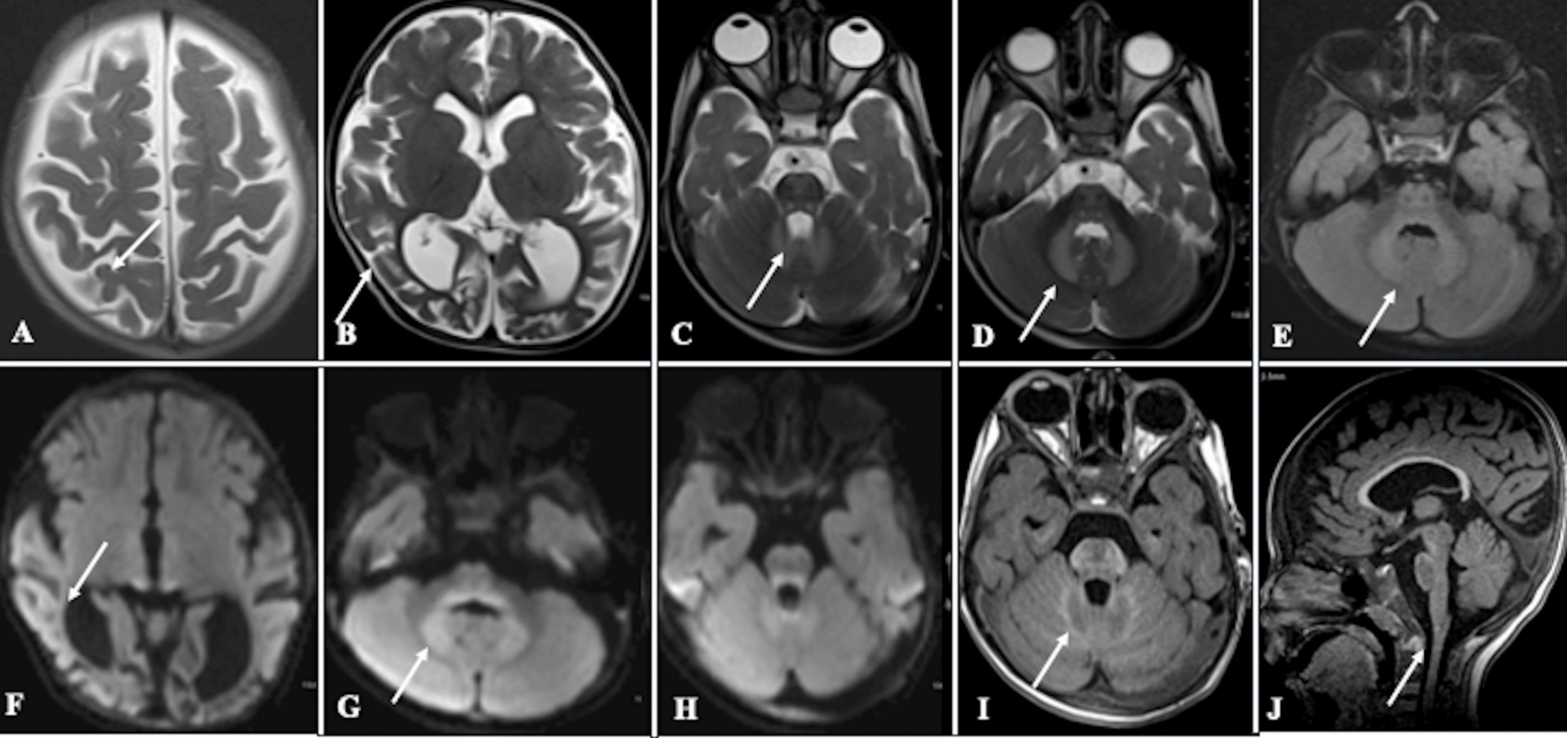
Magnetic resonance imaging (MRI) images of the brain showing the findings of EAST syndrome. Axial T2 (A-D) and Axial FLAIR (E) images showed cortical atrophy (white arrows in A, B) with hyperintense signal changes in cerebellar dentate nuclei, pons, and midbrain (white arrows in C-E) and restricted diffusion in the corresponding locations (white arrows in F-H). Axial T1 (I) showed the hypo intensity in the corresponding region (white arrow). Sagittal T1 (J) showed mild atrophy in the cervical spinal cord (white arrow). EAST: Epilepsy, Ataxia, Sensorineural Deafness, Tubulopathy Syndrome

On evaluation, he had urinary potassium of 48 meq/L in 24 hr, suggesting tubular loss. His serum magnesium and urinary calcium excretion were within normal limits. Because of potassium wasting with metabolic alkalosis, the possibility of Gettleman syndrome vs Barter syndrome was considered. Magnetic resonance imaging of the brain was done because of a seizure, which revealed diffuse cerebral atrophy, predominantly in the bilateral posterior parietal and occipital lobes. T2/FLAIR (fluid-attenuated inversion recovery) hyperintensity was seen in the medulla, midbrain, dorsal pons, and bilateral cerebellar dentate nuclei with diffusion restriction on DWI (diffusion-weighted imaging) sequences (Figure 1A-1H). Subtle T2/FLAIR hyperintensity with mild restricted diffusion was seen in bilateral parieto-occipital regions (Figure 1A, 1F). There was no cerebellar hypoplasia. The corpus callosum, brainstem, and visualized upper cervical spinal cord appeared atrophic (Figure 1J).

Brainstem evoked response audiometry (BERA) showed moderate sensory neural hearing loss. Findings were suggestive of EAST syndrome. He was started on a potassium supplement and amiloride, after which potassium was maintained at a low normal range. Seizures were controlled with antiseizure medication (levetiracetam, valproate, oxcarbamazepine). Parents were counselled regarding molecular testing for tubulopathy, which they denied due to financial constraints. On follow-up, the child was seizure-free, with potassium near normal. However, his milestones were delayed, and he was advised for physiotherapy.

## Discussion

EAST syndrome is a rare autosomal recessive disorder due to a mutation in the *KCNJ10 *gene on chromosome 1q23.2, encoding an ATP-sensitive inward rectifier potassium channel, Kir4.1. Clinical phenotypic heterogeneity correlates with the variable expression of this gene in the brain, ear, eye, and kidneys [4]. KCNJ10-related potassium channels are expressed in glial cells, responsible for the uptake of excess potassium in extra-cellular space, and redistributed via gap junctions into intracellular space. In this way, these channels play an essential role in removing and redistributing extra-cellular potassium and hence have a protective potassium siphoning mechanism [5]. This reduces the risk of excessive excitability of neurons, which leads to seizure activity. Any mutation in this gene will lead to the loss of this protective mechanism and hence explains epilepsy as the most consistent feature of EAST syndrome. In most of the documented cases, seizures have been described to be of generalized type; however, it is not clear whether these seizures can be of secondary generalization with focal onset [6]. In our case, also in the current presentation, at three years of age, he had a generalized tonic-clonic type of seizure semiology involving all limbs. Seizures in EAST syndrome patients are usually well controlled with the use of antiepileptic drugs. However, recurrence has also been described. Global developmental delay, predominantly motor milestones delay, has been observed in almost all patients. Ataxia becomes evident when the child starts walking without support if there is no developmental delay in achieving motor milestones till the first year of life. Hence, ataxia is an important clinical manifestation of EAST syndrome and can either remain static, non-progressive, or even progress with increasing age. However, in our case, there was a significant motor developmental delay, and the child was able to sit with support at the age of 3 years. Therefore, ataxia could not be elicited in our patient.

*KCNJ10 *gene expression in intermediate cells of stria vascularis of the inner ear has been described in mice, which is responsible for high potassium concentration in endolymph [7]. These channels facilitate potassium entry into the cochlea's hair cells, contributing to signal transduction. Mutation in the *KCNJ10 *gene will, therefore, result in varying degrees of hearing loss depending on the type of mutation involved. A varying degree of hearing impairment has been described in the literature, ranging from mild sensorineural hearing loss to severe hearing impairment, like our case, and many of them require a hearing aid in the first decade of their life [8]. Other conditions that can cause epilepsy, global developmental delay, and sensorineural hearing loss include congenital TORCH (Toxoplasma gondii, Other agents, Rubella, Cytomegalovirus, and Herpes simplex virus) infections or any hypoxic-ischemic insult during the perinatal period. But in our case, both the antenatal and perinatal periods were uneventful.

In renal distal convoluted tubules and collecting ducts, this gene expression has been described in the basolateral membrane. This distal part of the nephron is responsible for fluid and electrolyte homeostasis and contributes to maximum transport activity. *KCNJ10 *- potassium channels facilitate ion transport across distal nephron, including sodium influx and chloride efflux, which is mediated by Na+/K+-ATPase activity and pump-leak coupling [9]. *KCNJ10 *gene mutations in EAST syndrome patients will disrupt this ion transport activity and lead to loss of sodium chloride. This renal salt-wasting leads to renin-angiotensin and aldosterone system activation, increasing renin and aldosterone levels. Increased aldosterone will then activate amiloride-sensitive ENaC (epithelial sodium) channels located on distal convoluted tubules and the collecting system, resulting in sodium reabsorption from the distal nephron as a compensatory mechanism. This sodium reabsorption is accompanied by compensatory secretion of potassium and hydrogen ions into urine, ultimately leading to hypokalemic metabolic alkalosis. The present case had hypokalemic metabolic alkalosis at six months of age and was treated with potassium chloride supplementation; however, some improvement was seen after potassium chloride treatment. This salt-wasting distal renal tubular dysfunction phenotype can also be seen in other disorders like Gitelman and Barter syndromes; however, extra-renal manifestations are not seen in these patients, as in our case.

Neuroimaging findings in EAST syndrome have been described as completely normal in a few cases, to variable degrees of cerebellar hypoplasia in most patients [8]. Bilateral dentate nuclei signal changes are the second most common findings reported after cerebellar hypoplasia. Other less common but reported findings include brainstem and spinal cord atrophy, atrophic corpus callosum, and variable degrees of cerebral atrophy. Non-specific white matter signal alterations have also been reported in some cases. Diffusion restriction in the involved regions is consistent with intra-myelinic edema, seen due to disrupted ion-water homeostasis [10]. Similar imaging findings were observed in the present case without cerebellar atrophy.

## Conclusions

EAST syndrome is a rare disorder characterized by neurological, auditory, and renal involvement. Brain imaging, particularly targeting the characteristic sites of involvement, can aid in the diagnosis and is valuable for monitoring disease progression. Although the existing literature predominantly describes EAST syndrome as a non-progressive condition, the limited number of reported cases underscores the need for further research to confirm the long-term nature and variability of the disease.
